# *CYP2C9* polymorphism is associated with susceptibility to ischemic stroke in a Chinese population

**DOI:** 10.1080/07853890.2025.2579788

**Published:** 2025-10-30

**Authors:** Jinglu Zhang, Haiyu Jia, Yun Liu, Yong Zhang, Haihong Nie, Qiuhong Bao

**Affiliations:** Gerontological Center, The Affiliated Hospital of Inner Mongolia Medical University, Hohhot, China

**Keywords:** Ischaemic stroke, susceptibility, CYP2C9, polymorphism

## Abstract

**Objective:**

Ischemic stroke (IS) is a global cerebrovascular disease with high morbidity and mortality. *CYP2C9* genetic variants are significantly involved in the development of many diseases. However, the impacts that *CYP2C9* genetic variants on the risk of IS remain to be comprehensively understood. We aim to determine the association of *CYP2C9* with IS susceptibility in the Chinese Han population.

**Methods:**

This study recruited a total of 643 patients with IS and 643 healthy controls. *CYP2C9* single-nucleotide polymorphisms (SNPs) were tested by the MassARRAY iPLEX platform. The associations of *CYP2C9* polymorphisms (rs10509679, rs1934967, rs1934968, and rs9332220) and IS risk were analysed by the logistic regression analysis.

**Results:**

Our study showed that rs10509679 G > A was significantly related to increased risk of IS (OR= 1.48, 95% CI= 1.05–2.09, *p* = 0.024). The stratified analysis further demonstrated that rs10509679 was correlated with an increased susceptibility to IS patients in subgroups of people aged> 60 years, those with BMI< 24, smokers, drinkers, and those without diabetes. False-positive report probability analysis was further carried out to verify the significant findings. In haplotype analysis, individuals carrying the A_rs10509679_C_rs1934967_G_rs1934968_G_rs9332220_ haplotype exhibit an elevated risk of IS occurrence.

**Conclusion:**

In summary, *CYP2C9* rs10509679 G > A may be associated with the risk of IS.

## Introduction

Ischemic stroke (IS), commonly known as cerebral infarction, is a cerebrovascular disease caused by ischemia and hypoxia in local brain tissue due to cerebral vascular occlusion, which leads to brain cell death [1]. The main clinical features of IS are sudden local neurological dysfunction, such as limb weakness, slurred speech, facial deviation, etc [2]. The aetiology of IS is very complicated, including atherosclerosis, cardiogenic embolism, and vasculitis, with atherosclerosis being the most common cause [3]. Previous studies have demonstrated that some risk factors (like smoking, drinking, diabetes, obesity, hypertension, and hyperlipidaemia) can significantly increase the risk of IS [3,4]. Recently, it has been shown that genetic factors strongly contribute to the progress of IS. For example, some genetic variants may affect blood coagulation mechanisms and increase the risk of thrombosis [5,6]. Besides, genetic factors may also indirectly increase the risk of stroke by affecting blood pressure, blood lipids, and other physiological indicators [7,8]. Moreover, Deng et al. have demonstrated that *ERCC1* rs3212986 polymorphism is related to increased IS risk [9]. Wang et al. showed that *NQO1* rs2917673 was a risk factor for IS [10]. IS is extremely harmful, which not only leads to a serious decline in patients’ quality of life but also results in long-term disability and even death. Therefore, an in-depth exploration of the role of genetic factors in IS will not only help to reveal the pathogenesis of the disease but also give a scientific basis for the development of targeted gene therapy strategies, thereby offering patients more effective treatment options.

Cytochrome P450 2C9 (CYP2C9), the human cytochrome P450 enzyme family member, has a key role in various biochemical processes such as drug metabolism, hormone synthesis, and cholesterol synthesis [11]. *CYP2C9* is mainly expressed in the liver and plays a key role in the metabolism of various drugs, including non-steroidal anti-inflammatory drugs, anticoagulants, and antiepileptic drugs. *CYP2C9* influences the activity of antithrombotic medications such as warfarin and clopidogrel *via* metabolic activation. Genetic polymorphisms reduce enzymatic activity, leading to abnormal drug metabolism and increased risks of thrombosis or haemorrhage [12–15]. Additionally, *CYP2C9* polymorphisms are associated with enhanced vulnerability of atherosclerotic plaques and elevated risk of embolic stroke, potentially mediated by disrupted eicosanoid metabolism that exacerbates vascular inflammation and endothelial dysfunction [16,17]. Furthermore, *CYP2C9* activity is regulated by Nrf2, which indirectly modulates stroke risk during antithrombotic therapy through its influence on atrial fibrosis [18]. *CYP2C9* polymorphism is involved in many diseases’ occurrence and progression. Research showed that certain variants in the *CYP2C9* gene can accelerate the susceptibility of cardiovascular disease [12]. Cullell et al. reported the *CYP2C9* genetic polymorphisms may influence the metabolism of warfarin, thereby affecting its efficacy and the risk of bleeding [19]. Furthermore, multiple studies have suggested that *CYP2C9* may play a potential role in stroke susceptibility. For instance, a study conducted by Peng et al. in 2025 found that the *CYP2C9* rs2860905 variant significantly increased the risk of IS in the Taiwanese population [20]. Additionally, another study by Peng et al. demonstrated that the CYP2C9 rs4918758 variant significantly influenced the risk of IS in Taiwanese males [21]. In mainland China, research on the association between *CYP2C9* genetic polymorphisms and IS remains relatively limited, and comprehensive studies clarifying this relationship are still lacking. However, some studies have begun to explore the distribution of *CYP2C9* genetic polymorphisms across different regions of China and their impact on drug response. For example, a 2025 study found regional differences in the frequency of CYP2C9*2 and *3 alleles in Chinese populations, with these polymorphisms being closely associated with warfarin dose requirement [22]. From a global perspective, research on *CYP2C9* genetic polymorphisms indicates significant differences in their distribution across various ethnic and racial groups. For instance, the CYP2C9*2 and *3 alleles are more prevalent in European and Middle Eastern populations but relatively rare in East Asian and sub-Saharan African populations [22]. The impact of these polymorphisms on drug metabolism also varies among populations, underscoring the importance of conducting studies in diverse populations to advance the goals of precision medicine.

Thus, we conducted this case-control study to determine the association between *CYP2C9* polymorphisms with IS risk in a Chinese Han population. The study cohort comprised 643 IS patients and 643 healthy controls. Four candidate SNPs (rs10509679, rs1934967, rs1934968, and rs9332220) were selected as tag-SNPs based on the following criteria: minor allele frequency (MAF) >5% in CHB from the 1000 Genomes Project, min genotype > 75%, r^2^ < 0.8, and Hardy-Weinberg equilibrium (HWE) p-value > 0.05, and call rate >95%.

## Methods

### Study subjects

The sample size was determined using G*Power software (version 3.1.9.7). The calculation process proceeded as follows: First, we selected the t-test as the statistical method and specified the analysis type for ‘Difference between two independent means (two groups)’. Subsequently, parameters were configured with these settings: two-tailed test (tail = 2), effect size *d* = 0.2, significance level α = 0.05, statistical power (1-β) = 0.9475, and allocation ratio N2/N1 = 1. The calculation yielded a required sample size of 643 participants for both the case group and the control group. This research was approved by the ethics committee of the Affiliated Hospital of Inner Mongolia Medical University and all experiments were carried out following the principles of the 1964 Declaration of Helsinki. All participants were informed and signed a written informed consent form. In this project, we recruited 1286 unrelated adult subjects consisting of 643 IS patients and 643 healthy controls from the Affiliated Hospital of Inner Mongolia Medical University. The patients were newly diagnosed and confirmed as IS by two experienced neurologists based on clinical symptoms, physical examination, magnetic resonance imaging (MRI), and/or cranial computed tomography according to the diagnostic criteria for stroke [23]. The exclusion criteria for cases were: (1) age≤ 18 years old. (2) patients with a history of stroke. (3) patients with transient ischaemic attack and haemorrhagic stroke. (4) patients with cerebral vascular malformation. (5) patients with brain tumours. (6) patients with any type of disease, including cardiac, neurological, and autoimmune diseases. Controls were selected from healthy individuals who had a physical examination at the same hospital during the same period. Controls were matched to patients by gender. Individuals with hypertension and diabetes (including current and prior diagnoses) were excluded based on medical record review and structured health questionnaires. Additionally, participants with a family history of neurological or brain disorders were excluded. Demographic characteristics and environmental exposures, such as smoking and drinking status, for all participants were collected through medical records and health questionnaires.

### Polymorphism selection and genotyping

The selection criteria of the four *CYP2C9* gene SNPs (rs10509679, rs1934967, rs1934968, and rs9332220) were as follows: First, we extracted *CYP2C9* variants from the human GRCh37 reference genome (chromosome 10: 94,938,658-94,990,091) using VCF to PED Converter, obtaining 249 SNPs from the Chinese Han in Beijing (CHB) population. Subsequently, Haploview software was applied for quality control and tag-SNP selection, with thresholds set as minor allele frequency (MAF) > 0.05, min genotype > 75%, r^2^ < 0.8, and Hardy-Weinberg equilibrium (HWE) *p*-value > 0.05. Additionally, SNPs with call rates below 95% were excluded. The finalized SNPs were retained for analysis. Genomic DNA was extracted from peripheral blood samples of each participant using a Gold Purification Kit. Genotyping was performed using Agena MassARRAY iPLEX platform under the protocols of the manufacturer. Besides, the data of genotyping was analyzed using Agena Bioscience TYPER software.

### Bioinformatic analysis

The potential functions of each SNP were predicted through HaploReg V4.2 online software (https://pubs.broadinstitute.org/mammals/haploreg/haploreg.php).

### Statistical analyses

Fisher’s exact test was used to evaluate the Hardy–Weinberg equilibrium (HWE) of these SNPs in the controls. The associations between *CYP2C9* polymorphisms and IS risk were assessed using logistic regression analysis under five genetic models (allele, codominant, dominant, recessive, and log-additive), with adjustments for age, hypertension, and diabetes. Besides, we also investigated the associations stratified by age, sex, BMI, smoking, drinking, hypertension, and diabetes. The relative risk of IS was calculated by using adjusted odds ratios (ORs) and 95% confidence intervals (CIs). The genetic models are defined as follows (with A representing the wild-type allele and B the mutant allele): Allelic model: B allele vs. A allele (reference allele: A). Codominant model: BB vs. AA and AB vs. AA (reference group: AA). Dominant model: AB/BB vs. AA (reference group: AA). Recessive model: BB vs. AA/AB (reference group: AA/AB). Log-additive model: Trend effect per additional B allele (comparison: AA vs. AB vs. BB). The Benjamini–Hochberg false discovery rate (FDR) method was used to control for multiple testing. The false-positive report probability (FPRP) was carried out to validate the significant findings. Furthermore, Linkage disequilibrium (LD) was analyzed using Haploview V4.2 software, and haplotype analyses were conducted using the SNPStats online tool. Statistical analyses were conducted by SPSS 22.0 software. The chi-square test was used to detect the statistical difference of discrete variables, such as sex, drinking, and smoking status between the two groups. The difference in age between age and controls was compared by student’s *t*-test. The significance level was set at *p* < 0.05.

## Results

### Demographic characteristics

The clinical features are shown in Table 1. Our study included 643 patients with IS (427 men and 216 women) and 643 (430 men and 213 women) healthy controls. No significant difference was found in age between the cases and controls (*p* < 0.001). In terms of sex, BMI, drinking, and smoking status, no significant differences were observed (all *p* > 0.05).

**Table 1.** Clinical features of is patients and the controls.

Variables	Case group (*n* = 643)	Control group (*n* = 643)	*p*
Sex^a^			0.859
Men	427 (66.4%)	430 (66.9%)	
women	216 (33.6%)	213 (33.1%)	
Age, years (mean ± SD)^b^	63.57 ± 9.92	60.69 ± 6.03	< 0.001
> 60	411 (63.9%)	335 (52.1%)	
≤ 60	232 (36.1%)	308 (47.9%)	
Smoking^a^			0.577
Yes	322 (50.1%)	312 (48.5%)	
No	321 (49.9%)	331 (51.5%)	
Drinking^a^			0.823
Yes	328 (51.0%)	324 (50.4%)	
No	315 (49.0%)	319 (49.6%)	
BMI (kg/m^2^)^a^			0.200
≥ 24	217 (33.7%)	239 (37.2%)	
< 24	426 (66.3%)	404 (62.8%)	
Hypertension			
No	199 (30.9%)	643	
Yes	444 (69.1%)	0	
Diabetes			
Yes	110 (17.1%)	0	
No	533 (82.9%)	643	

^a^Pearson′s *X^2^* test is used. ^b^Student’s t-test is used. *p* < 0.05 indicates statistical significance.

IS, ischemic stroke.

### Association between CYP2C9 polymorphisms and is susceptibility

In this study, we detected four SNPs (rs10509679, rs1934967, rs1934968, and rs9332220) in the *CYP2C9* gene. Table 2 exhibited detailed information on the SNPs. The genotype distributions of these SNPs followed HWE in the controls (*p* = 0.845, *p* = 0.889, *p* = 0.935, and *p* = 0.645, respectively). The association of these SNPs with IS risk was analysed by logistic regression analysis, as depicted in Table 3. We found that rs10509679 was associated with an increased susceptibility to IS (A Vs G: OR= 1.21, 95% CI= 1.02–1.43, *p* = 0.030, *p* (FDR)= 0.120; GA Vs GG: OR= 1.47, 95% CI= 1.03–2.11, *p* = 0.034, *p* (FDR)= 0.135; GA-AA Vs GG: OR= 1.48, 95% CI= 1.05–1.70, *p* = 0.024, *p* (FDR)= 0.095).

**Table 2.** Basic characteristics of the candidate SNPs in the *CYP2C9* gene.

SNP ID	Chromosome position	Role	Alleles (A/B)	MAF		*p*-HWE	HaploReg v4.2
				Case	Control		
rs10509679	Chr10: 94948469	intron	A/G	0.321	0.282	0.845	Motifs changed, GRASP QTL hits, Selected eQTL hits
rs1934967	Chr10: 94981669	intron	T/C	0.161	0.170	0.889	Selected eQTL hits
rs1934968	Chr10: 94982060	intron	A/G	0.392	0.413	0.935	Motifs changed
rs9332220	Chr10: 94984186	intron	A/G	0.094	0.096	0.645	Enhancer histone marks, Motifs changed, Selected eQTL hits

SNP, single nucleotide polymorphisms; A, minor allele; B, major allele; MAF, minor allele frequency; HWE, Hardy–Weinberg equilibrium.

*p* < 0.05 are excluded.

**Table 3.** Association between *CYP2C9* gene polymorphisms and ischemic stroke susceptibility.

SNP ID	Model	Genotype	Case N	Control N	Without adjusted		With adjusted		
					OR (95% CI)	*p^a^*	OR (95% CI)	*p^b^*	*p* (FDR)
rs10509679	Allele	G (Ref.)	869	918	1 (Ref.)				
		A	411	360	**1.21 (1.02–1.43)**	**0.030**	/	/	0.120
	Codominant	G/G (Ref.)	287	331	1 (Ref.)		1 (Ref.)		
		G/A	295	256	**1.33 (1.06–1.67)**	**0.015**	**1.47 (1.03–2.11)**	**0.034**	0.135
		A/A	58	52	1.29 (0.86–1.93)	0.225	1.53 (0.84–2.79)	0.166	0.663
	Dominant	G/G (Ref.)	287	331	1 (Ref.)		1 (Ref.)		
		G/A-A/A	189	176	**1.32 (1.06–1.65)**	**0.013**	**1.48 (1.05–2.09)**	**0.024**	0.095
	Recessive	G/G-G/A (Ref.)	582	587	1 (Ref.)		1 (Ref.)		
		A/A	58	52	1.13 (0.76–1.66)	0.556	1.27 (0.72–2.25)	0.414	0.827
	Log-additive	–	–	–	**1.21 (1.02–1.44)**	**0.028**	**1.32 (1.02–1.70)**	**0.035**	0.827
rs1934967	Allele	C (Ref.)	1079	1064	1 (Ref.)				
		A	207	218	0.94 (0.76–1.15)	0.536	/	/	0.714
	Codominant	C/C (Ref.)	454	442	1 (Ref.)		1 (Ref.)		
		C/A	171	180	0.92 (0.72–1.18)	0.535	0.89 (0.61–1.31)	0.568	1.136
		A/A	18	19	0.92 (0.48–1.78)	0.810	0.81 (0.27–2.45)	0.711	0.711
	Dominant	C/C (Ref.)	454	442	1 (Ref.)		1 (Ref.)		
		C/A-A/A	189	199	0.92 (0.73–1.17)	0.519	0.89 (0.61–1.29)	0.527	0.702
	Recessive	C/C-C/A (Ref.)	625	622	1 (Ref.)		1 (Ref.)		
		A/A	18	19	0.94 (0.49–1.81)	0.860	0.84 (0.28–2.51)	0.753	0.753
	Log-additive	–	–	–	0.94 (0.76–1.15)	0.538	0.90 (0.64–1.25)	0.514	0.753
rs1934968	Allele	A (Ref.)	782	749	1 (Ref.)				
		C	504	527	0.92(0.78–1.07)	0.276	/	/	0.553
	Codominant	A/A (Ref.)	241	219	1 (Ref.)		1 (Ref.)		
		A/C	300	311	0.88 (0.69–1.12)	0.286	0.93 (0.64–1.34)	0.682	0.909
		C/C	102	108	0.86 (0.62–1.19)	0.359	0.77 (0.46–1.29)	0.320	0.639
	Dominant	A/A (Ref.)	241	219	1 (Ref.)		1 (Ref.)		
		A/C-C/C	402	419	0.87 (0.69–1.10)	0.239	0.88 (0.62–1.26)	0.493	0.986
	Recessive	A/A-A/C (Ref.)	541	530	1 (Ref.)		1 (Ref.)		
		C/C	102	108	0.93 (0.69–1.24)	0.607	0.80 (0.50–1.29)	0.365	1.462
	Log-additive	–	–	–	0.92 (0.78–1.07)	0.278	0.89 (0.69–1.13)	0.339	1.462
rs9332220	Allele	T (Ref.)	1163	278	1 (Ref.)				
		A	121	264	0.98(0.76–1.28)	0.903	/	/	0.903
	Codominant	T/T (Ref.)	1163	278	1 (Ref.)		1 (Ref.)		
		T/A	121	264	1.06 (0.80–1.42)	0.687	0.91 (0.57–1.45)	0.704	0.704
		A/A	524	527	0.43 (0.11–1.68)	0.225	0.50 (0.06–4.13)	0.520	0.694
	Dominant	T/T (Ref.)	115	109	1 (Ref.)		1 (Ref.)		
		T/A-A/A	3	7	1.02 (0.77–1.36)	0.875	0.89 (0.56–1.40)	0.608	0.608
	Recessive	T/T-T/A (Ref.)	524	527	1 (Ref.)		1 (Ref.)		
		A/A	118	116	0.43 (0.11–1.66)	0.219	0.51 (0.06–4.18)	0.529	0.705
	Log-additive	–	–	–	0.98 (0.75–1.28)	0.902	0.87 (0.57–1.33)	0.531	0.705

OR, odds ratio, 95 % CI; 95% confidence intervals, Ref., reference. The *p^a^*‐values were calculated by unconditional logistic regression analysis without adjustment. The *p^b^* values were calculated by logistic regression adjusted by age, hypertension, and diabetes. The threshold value for FDR was 0.05. Bold text represents statistical significance.

### Stratification analysis of significant SNPs

We further assessed the relationship between *CYP2C9* polymorphisms and the risk of IS stratified by age, sex, BMI, smoking, drinking, and diabetes. As shown in Table 4, our study revealed that rs10509679 was significantly related to IS risk among individuals aged> 60 years (OR= 1.23, 95% CI= 1.03–1.61, *p* = 0.027, *p* (FDR)= 0.109; GA Vs GG: OR= 1.61, 95% CI= 1.02–2.53, *p* = 0.042, *p* (FDR)= 0.167; AG-AA Vs GG: OR= 1.59, 95% CI= 1.03–2.45, *p* = 0.037, *p* (FDR)= 0.150), women (OR= 1.40, 95% CI= 1.04–1.88, *p* = 0.027, *p* (FDR)= 0.106), and BMI< 24 (OR= 1.30, 95% CI= 1.05–1.61, *p* = 0.016, *p* (FDR)= 0.064; AG-AA Vs GG: OR= 1.54, 95% CI= 1.02–2.33, *p* = 0.041, *p* (FDR)= 0.165). Furthermore, rs10509679 showed an increased risk for IS patients in the subgroups of smokers (OR= 1.37, 95% CI= 1.08–1.74, *p* = 0.011, *p* (FDR)= 0.043; GA Vs GG: OR= 1.77, 95% CI= 1.06–2.96, *p* = 0.030, *p* (FDR)= 0.120; AG-AA Vs GG: OR= 1.85, 95% CI= 1.14–3.02, *p* = 0.013, *p* (FDR)= 0.045), drinkers (OR= 1.36, 95% CI= 1.07–1.72, *p* = 0.013, *p* (FDR)= 0.049; GA Vs GG: OR= 1.77, 95% CI= 1.09–2.89, *p* = 0.022, *p* (FDR)= 0.089; AG-AA Vs GG: OR= 1.78, 95% CI= 1.11–2.84, *p* = 0.016, *p* (FDR)= 0.064), and individuals without diabetes (OR= 1.27, 95% CI= 1.06–1.51, *p* = 0.008, *p* (FDR)= 0.034; GA Vs GG: OR= 1.47, 95% CI= 1.03–2.11, *p* = 0.034, *p* (FDR)= 0.135; AG-AA Vs GG: OR= 1.48, 95% CI= 1.05–2.09, *p* = 0.024, *p* (FDR)=) (Table 5).

**Table 4.** Stratified analysis between rs10509679 polymorphism and the risk of ischemic stroke.

Allele/Genotype	Without adjusted		With adjusted			Without adjusted		With adjusted			*p*-interaction
	OR (95% CI)	*p^a^*	OR (95% CI)	*p^b^*	*p* (FDR)	OR (95% CI)	*p^b^*	OR (95% CI)	*p^b^*	*p* (FDR)	
	**Age> 60 years**					**Age≤ 60 years**					
G (Ref.)	1 (Ref.)					1 (Ref.)					
A	**1.23 (1.03–1.61)**	**0.027**	/	/	0.109	1.11 (0.86–1.45)	0.419	**/**	**/**	0.839	
GG (Ref.)	1 (Ref.)		1 (Ref.)			1 (Ref.)		1 (Ref.)			
AG	**1.51 (1.11–2.05)**	**0.009**	**1.61(1.02–2.53)**	**0.042**	0.167	1.16 (0.81–1.65)	0.429	1.36 (0.75–2.46)	0.314	1.223	0.82
AA	1.34 (0.79–2.28)	0.276	1.51 (0.70–3.24)	0.297	1.188	1.20 (0.63–2.28)	0.582	1.50 (0.55–4.14)	0.429	1.382	
GG (Ref.)	1 (Ref.)		1 (Ref.)			1 (Ref.)		1 (Ref.)			
AG-AA	**1.48 (1.10–1.98)**	**0.009**	**1.59 (1.03–2.45)**	**0.037**	0.150	1.16 (0.82–1.64)	0.391	1.38 (0.78–2.44)	0.268	1.053	0.67
GG-AG (Ref.)	1 (Ref.)		1 (Ref.)			1 (Ref.)		1 (Ref.)			
AA	1.11 (0.67–1.85)	0.684	1.21 (0.58–2.51)	0.616	1.232	1.12 (0.60–2.08)	0.725	1.29 (0.49–3.38)	0.603	1.744	0.78
	**Men**					**Women**					
G (Ref.)	1 (Ref.)					1 (Ref.)					
A	1.12 (0.91–1.38)	0.273	/	/	1.091	**1.40 (1.04–1.88)**	**0.027**	/	/	0.106	
GG (Ref.)	1 (Ref.)		1 (Ref.)			1 (Ref.)		1 (Ref.)			
AG	1.20 (0.90–1.58)	0.213	1.44 (0.93–2.25)	0.105	0.420	**1.65 (1.10–2.47)**	**0.015**	1.47 (0.79–2.70)	0.221	0.442	0.99
AA	1.17 (0.71–1.93)	0.548	1.43 (0.68–3.03)	0.344	1.378	1.55 (0.78–3.11)	0.214	1.54 (0.55–4.28)	0.412	0.825	
GG (Ref.)	1 (Ref.)		1 (Ref.)			1 (Ref.)		1 (Ref.)			
AG-AA	1.19 (0.91–1.56)	0.203	1.44 (0.94–2.21)	0.092	0.367	**1.63 (1.11–2.39)**	**0.012**	1.48 (0.83–2.65)	0.188	0.377	0.91
GG-AG (Ref.)	1 (Ref.)		1 (Ref.)			1 (Ref.)		1 (Ref.)			
AA	1.07 (0.66–1.74)	0.781	1.19 (0.59–2.42)	0.627	1.255	1.24 (0.63–2.41)	0.536	1.29 (0.48–3.45)	0.610	1.220	0.94
	**BMI< 24**					**BMI≥ 24**					
G (Ref.)	1 (Ref.)					1 (Ref.)					
A	**1.30 (1.05–1.61)**	**0.016**	/	/	0.064	1.07 (0.81–1.42)	0.625	**/**	**/**	2.500	
GG (Ref.)	1 (Ref.)		1 (Ref.)			1 (Ref.)		1 (Ref.)			
GA	**1.36 (1.02–1.80)**	**0.037**	1.48 (0.96–2.28)	0.075	0.300	1.28 (0.87–1.89)	0.215	1.43 (0.75–2.72)	0.278	1.110	0.70
AA	1.66 (0.96–2.86)	0.068	1.90 (0.89–4.06)	0.098	0.392	0.94 (0.50–1.77)	0.859	1.29 (0.47–3.55)	0.629	1.258	
GG (Ref.)	1 (Ref.)		1 (Ref.)			1 (Ref.)		1 (Ref.)			
GA-AA	**1.40 (1.06–1.84)**	**0.017**	**1.54 (1.02–2.33)**	**0.041**	0.165	1.20 (0.83–1.74)	0.323	1.40 (0.76–2.58)	0.283	1.132	0.72
GG-GA (Ref.)	1 (Ref.)		1 (Ref.)			1 (Ref.)		1 (Ref.)			
AA	1.44 (0.85–2.44)	0.177	1.58 (0.76–3.26)	0.221	0.462	0.84 (0.46–1.54)	0.574	1.08 (0.41–2.83)	0.873	1.350	0.40

OR, odds ratio, 95 % CI; 95% confidence intervals, Ref., reference. The *p^a^*‐values were calculated by unconditional logistic regression analysis without adjustment. The *p^b^* values were calculated by logistic regression adjusted by age, hypertension, and diabetes. The threshold value for FDR was 0.05. Bold text represents statistical significance.

**Table 5.** Association of rs10509679 polymorphism and ischemic stroke susceptibility stratified by smoking, drinking, hypertension, and diabetes.

Allele/Genotype	Without adjusted		With adjusted			Without adjusted		With adjusted			
	OR (95% CI)	*p^a^*	OR (95% CI)	*p^b^*	*p* (FDR)	OR (95% CI)	*p^a^*	OR (95% CI)	*p^b^*	*p* (FDR)	*p*-interaction
	**Smoking**					**Non-smoking**					
G (Ref.)	1 (Ref.)					1 (Ref.)					
A	**1.37 (1.08–1.74)**	**0.011**	/	/	**0.043**	1.07 (0.84–1.35)	0.603	**/**	**/**	2.412	
GG (Ref.)	1 (Ref.)		1 (Ref.)			1 (Ref.)		1 (Ref.)			
AG	**1.59 (1.14–2.21)**	**0.006**	**1.77 (1.06–2.96)**	**0.030**	0.120	1.13 (0.82–1.55)	0.472	1.24 (0.76–2.04)	0.394	1.574	0.36
AA	1.55 (0.88–2.71)	0.129	2.22 (0.91–4.90)	0.428	1.256	1.05 (0.58–1.90)	0.867	0.94 (0.36–2.46)	0.902	0.902	
GG (Ref.)	1 (Ref.)		1 (Ref.)			1 (Ref.)		1 (Ref.)			
AG-AA	**1.58 (1.15–2.16)**	**0.004**	**1.85 (1.14–3.02)**	**0.013**	**0.045**	1.11 (0.82–1.52)	0.494	1.19 (0.74–1.93)	0.471	1.882	0.21
GG-AG (Ref.)	1 (Ref.)		1 (Ref.)			1 (Ref.)		1 (Ref.)			
AA	1.25 (0.73–2.14)	0.422	1.70 (0.81–3.57)	0.164	0.656	0.99 (0.56–1.76)	0.984	0.85 (0.34–2.15)	0.727	0.727	0.29
	**Drinking**					**Non-drinking**					
G (Ref.)	1 (Ref.)					1 (Ref.)					
A	**1.36 (1.07–1.72)**	**0.013**	/	/	**0.049**	1.07 (0.84–1.36)	0.564	/	/	0.752	
GG (Ref.)	1 (Ref.)		1 (Ref.)			1 (Ref.)		1 (Ref.)			
AG	**1.62 (1.17–2.25)**	**0.004**	**1.77 (1.09–2.89)**	**0.022**	0.089	1.09 (0.78–1.51)	0.625	1.22 (0.72–2.05)	0.465	0.930	0.59
AA	1.45 (0.81–2.58)	0.210	1.81 (0.80–4.10)	0.154	0.308	1.14 (0.64–2.02)	0.652	1.28 (0.53–3.11)	0.588	2.353	
GG (Ref.)	1 (Ref.)		1 (Ref.)			1 (Ref.)		1 (Ref.)			
AG-AA	**1.59 (1.17–2.17)**	**0.003**	**1.78 (1.11–2.84)**	**0.016**	0.064	1.09 (0.80–1.50)	0.572	1.23 (0.74–2.02)	0.424	0.849	0.30
GG-AG (Ref.)	1 (Ref.)		1 (Ref.)			1 (Ref.)		1 (Ref.)			
AA	1.16 (0.66–2.02)	0.611	1.38 (0.63–3.00)	0.417	0.834	1.10 (0.63–1.90)	0.742	1.16 (0.50–2.72)	0.727	2.908	0.78
	**Diabetes**					**Non-diabetes**					
G (Ref.)	1 (Ref.)					1 (Ref.)					
A	0.93 (0.68–1.29)	0.680	**/**	**/**	2.720	**1.27 (1.06–1.51)**	**0.008**	**/**	**/**	**0.034**	
GG (Ref.)	1 (Ref.)		1 (Ref.)			1 (Ref.)		1 (Ref.)			
GA	1.00 (0.66–1.53)	0.988	1.73 (0.72–4.15)	0.217	0.870	**1.41 (1.11–1.80)**	**0.005**	**1.47 (1.03–2.11)**	**0.034**	0.135	NA
AA	0.77 (0.33–1.77)	0.537	0.68 (0.08–5.54)	0.722	1.444	1.42 (0.93–2.16)	0.105	1.53 (0.84–2.79)	0.166	0.663	
GG (Ref.)	1 (Ref.)		1 (Ref.)			1 (Ref.)		1 (Ref.)			
GA-AA	0.96 (0.64–1.45)	0.857	1.55 (0.66–3.65)	0.317	1.267	**1.41 (1.12–1.78)**	**0.003**	**1.48 (1.05–2.09)**	**0.024**	0.095	NA
GG-GA (Ref.)	1 (Ref.)		1 (Ref.)			1 (Ref.)		1 (Ref.)			
AA	0.77 (0.34–1.74)	0.525	0.52 (0.07–4.02)	0.535	2.138	1.20 (0.80–1.80)	0.373	1.27 (0.72–2.25)	0.414	0.827	1.00

OR, odds ratio, 95 % CI; 95% confidence intervals, Ref., reference. The *p^a^*‐values were calculated by unconditional logistic regression analysis without adjustment. The *p^b^* values were calculated by logistic regression adjusted by age, hypertension, and diabetes. The threshold value for FDR was 0.05. Bold text represents statistical significance.

### Haplotype analysis

**Figure 1.**
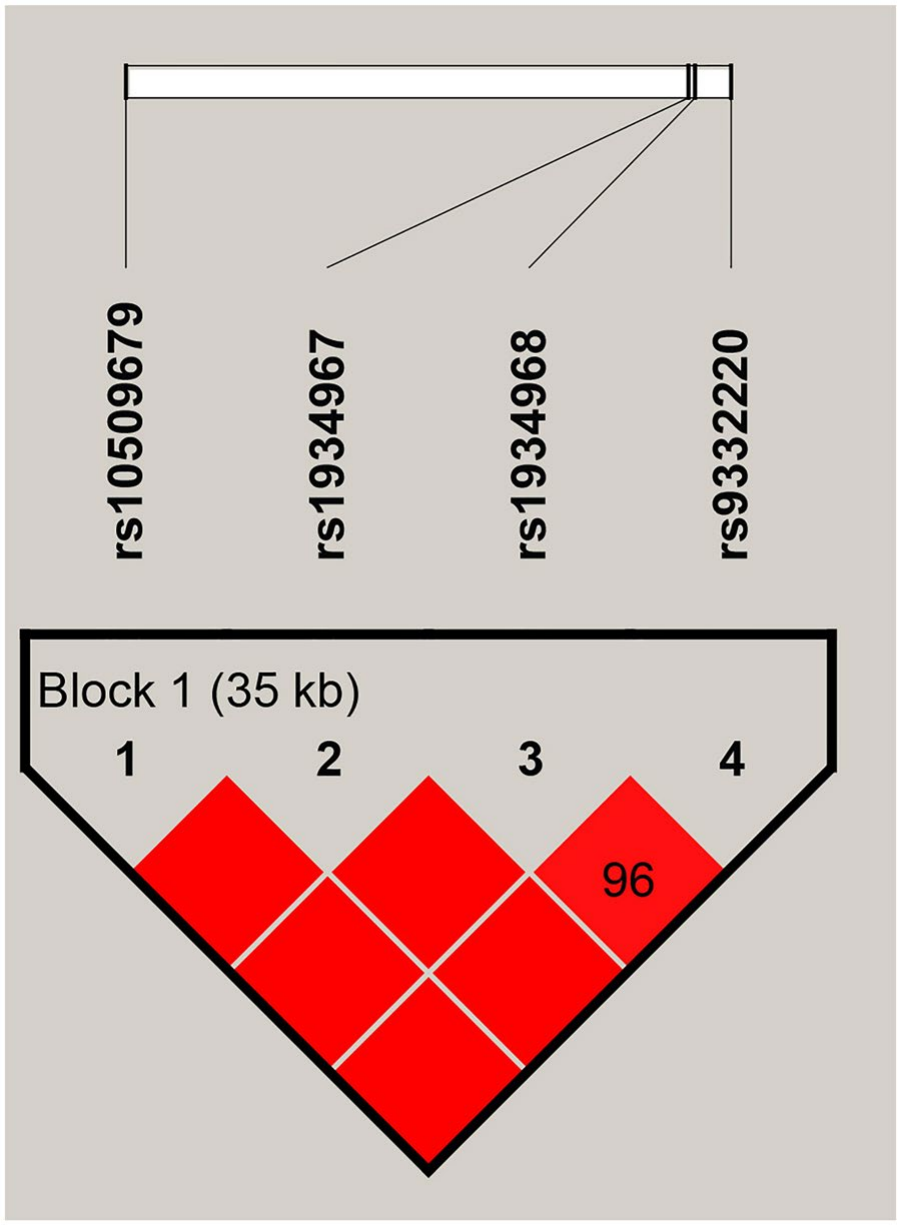
Haplotype Block Map of SNPs in *CYP2C9*. The numbers inside the diamonds indicate the pairwise D’.

We used Haploview software to analyze linkage disequilibrium (LD) in *CYP2C9* variants. A strong LD block spanning 35 kb was identified, encompassing rs10509679, rs1934967, rs1934968, and rs9332220 (Figure 1). Additionally, we performed a haplotype analysis to assess the combined effects of four *CYP2C9* SNPs. As shown in Table 6, the A_rs10509679_C_rs1934967_G_rs1934968_G_rs9332220_ haplotype was significantly associated with an increased risk of IS compared to the reference haplotype G_rs10509679_C_rs1934967_A_rs1934968_G_rs9332220_ (OR= 1.21, 95% CI: 1.00–1.46, *p* = 0.045).

**Table 6.** The haplotype analysis of *CYP2C9* gene polymorphisms.

Haplotypes	OR (95% CI)	*p*
G_rs10509679_C_rs1934967_A_rs1934968_G_rs9332220_	1 (reference)	
A_rs10509679_C_rs1934967_G_rs1934968_G_rs9332220_	**1.21 (1.00–1.46)**	**0.045**
G_rs10509679_T_rs1934967_G_rs1934968_G_rs9332220_	0.99 (0.80–1.24)	0.960
G_rs10509679_C_rs1934967_G_rs1934968_A_rs9332220_	1.06 (0.80–1.41)	0.670
G_rs10509679_C_rs1934967_G_rs1934968_G_rs9332220_	0.95 (0.61–1.49)	0.830

SNP: Single nucleotide polymorphism; 95% CI: 95% confidence interval; OR: Odds ratio.

Bold text represents statistical significance.

### FPRP result

We set the FPRP threshold at 0.2. As indicated in Table S1, the positive results for the rs10509679 remained noteworthy in the whole group and subgroups at the prior probability of 0.25 (FPRP< 0.2).

## Discussion

Stroke is a global cerebrovascular disease with high morbidity and mortality. Approximately 1.5 million people die from stroke each year in China, and 2.5 million new cases have been reported [24]. Stroke is mainly divided into IS and haemorrhagic stroke, and IS accounts for 87% of stroke cases [25]. The pathogenesis of IS involves multiple factors, including traditional risk factors and genetic factors [26,27]. *CYP2C9* plays an important role in IS progression. However, the impact of *CYP2C9* polymorphisms on IS susceptibility is unclear. Thus, this study explored the association of *CYP2C9* polymorphisms with IS risk. Our study demonstrated that rs10509679 polymorphism is related to an increased risk of IS. These findings may serve as potential genetic markers for IS risk assessment, facilitating early diagnosis, personalized treatment, and the development of prevention strategies for IS.

In this study, we observed that *CYP2C9* rs10509679 was significantly associated with an increased risk of IS. However, Zhang et al. did not find a significant correlation between rs10509679 and lung cancer [28], which may be attributed to differences in genetic background, sample size, or disease heterogeneity across study populations. Age, sex, BMI, smoking, drinking, and diabetes were risk factors in the development of IS [29–31]. To further explore the relationship between CYP2C9 polymorphism and IS susceptibility, we conducted a stratified analysis based on age, sex, BMI, smoking, drinking, and diabetes. Our study revealed that the association between rs10509679 and IS risk was more pronounced in subgroups of individuals aged> 60 years, those with BMI< 24, smokers, drinkers, and those without diabetes. These findings suggest that the impact of the *CYP2C9* rs10509679 polymorphism on IS risk may be modulated by environmental and clinical factors such as age, BMI, smoking, alcohol consumption, and diabetes. The result of the stratified analysis suggest that gene-environment interactions may play a crucial role in the occurrence of IS. For instance, individuals aged> 60 years may experience a heightened impact of CYP2C9 polymorphism on IS risk due to exacerbated metabolic dysfunction or increased oxidative stress. Individuals with a BMI< 24 may have lower levels of inflammation, which makes the potential impact of *CYP2C9* polymorphisms on endothelial function more pronounced. Additionally, smoking and alcohol consumption may further enhance the susceptibility to IS risk by increasing oxidative stress and inflammatory responses, amplifying the effect of CYP2C9 polymorphisms. The absence of diabetes may reflect the regulatory role of metabolic status on CYP2C9 function, which warrants further investigation.

It is noteworthy that other studies also support the role of gene–environment interactions in IS risk. For example, Liu et al. showed that miR-122 rs17669 was associated with risk of IS in non-drinker and men [32]. Additionally, *CYP4F2* rs2108622 exhibited a significant association in those aged> 60 years, those with BMI≥ 24, smokers, and drinkers [33]. Additionally, Huang et al. demonstrated that *CYP19A1* rs28757157 and rs3751591 polymorphisms conferred an increased susceptibility to IS in individuals aged> 60 years, drinkers, and smokers [34]. Besides, it was found that *CDH13* rs7193788 had higher risks for IS in patients with diabetes [35]. Taken above, we hypothesize that genetic susceptibility to IS may be regulated by age, sex, BMI, smoking, drinking, and diabetes, highlighting the significance of considering individual heterogeneity when exploring the relationship of genetic factors with IS risk. Future studies should further investigate how these factors regulate the genetic susceptibility to IS and consider the impact of environmental factors.

The rs10509679 polymorphism resides within an intron of *CYP2C9*. Although there have been no specific studies on the function of rs10509679 polymorphism, existing studies show that genetic variation within introns can affect gene expression through changing splice sites or affecting mRNA stability [36–38]. Here, we predicted the potential functions of rs10509679 through bioinformatic analysis. We found that rs10509679 was involved in the regulation of Selected eQTL hits, Motifs changed, and GRASP QTL hits. Future studies will verify the functional effects of rs10509679 on the *CYP2C9* gene expression and explore their association with IS risk.

This study has certain limitations, First, the study individuals were restricted to the Han population, which limits the generalizability of our findings to other ethnic groups. Further studies will include more diverse ethnic groups to improve the generalizability of the findings. Second, to more fully understand the functional significance of the *CYP2C9* polymorphisms, further studies is needed to validate the biological relevance of the findings through functional experiments.

## Conclusion

In summary, our study demonstrated that *CYP2C9* rs10509679 polymorphism may be associated with a higher risk of IS in the Chinese population. These findings contribute to our deeper understanding of how genetic variation influences IS risk.

## Supplementary Material

supplemental_tables.docx

## Data Availability

The data that support the findings of this study are available from the corresponding author upon reasonable request.
