# Supplementary material for: CYP2C9 polymorphism is associated with susceptibility to ischemic stroke in a Chinese population
Source: Ann Med. 2025 Oct 30;57(1):2579788. doi: 10.1080/07853890.2025.2579788 (PMC12576905; doi:10.1080/07853890.2025.2579788)
Supplement: supplemental_tables.docx [file IANN_A_2579788_SM6504.docx]

Table S1 False-positive report probability analysis for the positive findings between rs10509679 polymorphism and ischemic stroke risk

| Genotype and Variables | OR (95 % CI) | *p* Value | Statistical Power ^a^ | Prior Probability | | | |  |
| --- | --- | --- | --- | --- | --- | --- | --- | --- |
|  |  |  |  | 0.25 | 0.1 | 0.01 | 0.001 | 0.0001 |
| A Vs G | 1.21(1.02-1.43) | 0.030 | 1.000 | 0.071 ^b^ | 0.186 ^b^ | 0.715 | 0.962 | 0.996 |
| GA Vs GG | 1.47 (1.03-2.11) | 0.034 | 0.953 | 0.104 ^b^ | 0.257 | 0.792 | 0.975 | 0.997 |
| GA-AA Vs GG | 1.48 (1.05-2.09) | 0.024 | 0.956 | 0.075 ^b^ | 0.196 ^b^ | 0.729 | 0.964 | 0.996 |
| **Age> 60 years** |  |  |  |  |  |  |  |  |
| A Vs G | 1.23 (1.03-1.61) | 0.027 | 1.000 | 0.283 | 0.543 | 0.929 | 0.992 | 0.999 |
| AG Vs GG | 1.61(1.02-2.53) | 0.042 | 0.827 | 0.124 ^b^ | 0.298 | 0.823 | 0.979 | 0.998 |
| AG-AA Vs GG | 1.59 (1.03-2.45) | 0.037 | 0.851 | 0.111 ^b^ | 0.273 | 0.805 | 0.977 | 0.998 |
| **Women** |  |  |  |  |  |  |  |  |
| A Vs G | 1.40 (1.04-1.88) | 0.027 | 0.991 | 0.071 ^b^ | 0.187 ^b^ | 0.716 | 0.962 | 0.996 |
| **BMI< 24** |  |  |  |  |  |  |  |  |
| A Vs G | 1.30 (1.05-1.61) | 0.016 | 1.000 | 0.046 ^b^ | 0.127 ^b^ | 0.616 | 0.942 | 0.994 |
| GA-AA Vs GG | 1.54 (1.02-2.33) | 0.041 | 0.892 | 0.121 ^b^ | 0.293 | 0.820 | 0.979 | 0.998 |
| **Smoking** |  |  |  |  |  |  |  |  |
| A Vs G | 1.37 (1.08-1.74) | 0.011 | 0.999 | 0.029 ^b^ | 0.082 ^b^ | 0.494 | 0.908 | 0.990 |
| GA Vs GG | 1.77 (1.06-2.96) | 0.030 | 0.679 | 0.115 ^b^ | 0.281 | 0.811 | 0.977 | 0.998 |
| GA-AA Vs GG | 1.85 (1.14-3.02) | 0.013 | 0.622 | 0.063 ^b^ | 0.167 ^b^ | 0.688 | 0.957 | 0.996 |
| **Drinking** |  |  |  |  |  |  |  |  |
| A Vs G | 1.36 (1.07-1.72) | 0.013 | 0.999 | 0.030 ^b^ | 0.085 ^b^ | 0.505 | 0.911 | 0.990 |
| GA Vs GG | 1.77 (1.09-2.89) | 0.022 | 0.687 | 0.089 ^b^ | 0.227 | 0.764 | 0.970 | 0.997 |
| GA-AA Vs GG | 1.78 (1.11-2.84) | 0.016 | 0.688 | 0.064 ^b^ | 0.169 ^b^ | 0.691 | 0.958 | 0.996 |
| **Non-diabetes** |  |  |  |  |  |  |  |  |
| A Vs G | 1.27 (1.06-1.51) | 0.008 | 1.000 | 0.020 ^b^ | 0.058 ^b^ | 0.402 | 0.872 | 0.986 |
| GA Vs GG | 1.47 (1.03-2.11) | 0.034 | 0.953 | 0.104 ^b^ | 0.257 | 0.792 | 0.975 | 0.997 |
| GA-AA Vs GG | 1.48 (1.05-2.09) | 0.024 | 0.956 | 0.075 ^b^ | 0.196 ^b^ | 0.729 | 0.964 | 0.996 |

Statistical power ^a^ was calculated using the number of observations in the subgroup and the OR and *p* values in this table.

^b^ The level of false-positive report probability threshold was set at 0.2 and noteworthy findings are presented.
